# A frameshift insertion in *SGK3* leads to recessive hairlessness in Scottish Deerhounds: a candidate gene for human alopecia conditions

**DOI:** 10.1007/s00439-019-02005-9

**Published:** 2019-03-29

**Authors:** Marjo K. Hytönen, Hannes Lohi

**Affiliations:** 1 Department of Veterinary Biosciences, University of Helsinki, Helsinki, Finland; 2 Department of Medical Genetics, University of Helsinki, Helsinki, Finland; 3 The Folkhälsan Research Center, Helsinki, Finland

## Abstract

Hairlessness is a breed-specific feature selected for in some dog breeds but a rare abnormality in some others such as Scottish Deerhounds (SD). In SDs, the affected puppies are born with sparse hair but lose it within the first 2 months leaving the dogs completely hairless. The previous studies have implicated variants in *FOXI3* and *SGK3* in hairlessness; however, the known variants do not explain hairlessness in all breeds such as SDs. We investigated the genetic cause in 66 SDs, including a litter with two hairless dogs. We utilized a combined approach of genome-wide homozygosity mapping and whole-genome sequencing of a hairless SD followed by recessive filtering according to a recessive model against 340 control genomes. Only two homozygous-coding variants were discovered in the homozygosity regions, including a 1-bp insertion in exon 2 of *SGK3*. This results in a predicted frameshift and very early truncation (49/490 amino acids) of the SGK3 protein. Additional screening of the recessive variant demonstrated a full segregation with the hairlessness and a 12% carrier frequency in the SD breed. The variant was not found in the related Irish Wolfhound breed. This study identifies the second hairless variant in the *SGK3* gene in dogs and further highlights its role as a candidate gene for androgen-independent hair loss or alopecia in human.

**Electronic supplementary material:**

The online version of this article (10.1007/s00439-019-02005-9) contains supplementary material, which is available to authorized users.

## Introduction

Purebred dog breeds exhibit numerous different coat types and some breeds lack the coat altogether. The Chinese Crested and Peruvian and Mexican Hairless dogs are bred for almost hairless appearance. They have sparse or no body hair, but varying amounts of hair on the head, the tip of the tail, and distal parts of legs are allowed. These hairless dogs commonly have abnormal dentition (oligodontia and misshapen teeth) and occasionally malformations of the outer ear and auditory canal, as well (Drögemüller et al. 2008; Kupczik et al. 2017). Therefore, the trait selected in these breeds represents a medical condition, namely, ectodermal dysplasia. The trait is dominant and embryonic lethal as homozygous. It is caused by a 7-bp duplication, which results in a frameshift and a premature stop codon in the forkhead box I3 (*FOXI3*) gene (Drögemüller et al. 2008). The association of the FOXI3 transcription factor in canine ectodermal dysplasia embarked further studies to implicate its crucial functions during the development of the ectodermal organs such as hair follicle and teeth across species (Shirokova et al. 2013; Jussila et al. 2015; Shirokova et al. 2016; Birol et al. 2016). A member of the same gene family, *FOXI2*, has also been recently suggested to contribute to ectodermal dysplasia (Kurban et al. 2017).

Another type of hairless breed is American Hairless Terrier (AHT), where puppies are born with sparse hair that is permanently lost in a few weeks after the birth. The AHT breed was formed by purportedly selecting spontaneously hairless dogs from the Rat Terrier breed. The hairlessness in AHTs is an autosomal recessive trait caused by a 4-bp deletion in the serum/glucocorticoid regulated kinase family member 3 (*SGK3*) gene (Parker et al. 2017). The deletion in exon 4 results in a frameshift and premature stop codon removing the STKc_SGK3 catalytic domain of the protein.

Hairless dogs have been reported also in Scottish Deerhounds (SD) as an undesired phenotype with unknown genetic background. SD is an old sighthound breed originating from Scotland where it was used for hunting red deer until the end of nineteenth century before becoming a show breed. Our aim here was to identify the genetic cause and we describe the second hairless allele in *SGK3*, which represents a candidate gene for congenital human hair loss or alopecia.

## Materials and methods

### Study cohort and DNA extraction

EDTA blood samples were collected for DNA isolation from 66 Scottish Deerhounds (2 affected and 64 unaffected dogs) and 91 unaffected Irish Wolfhounds (IW). All dogs were privately owned dogs and the samples were collected with the dog owners’ informed consent. Genomic DNA was extracted from white blood cells using a semi-automated Chemagen extraction robot (PerkinElmer Chemagen Technologie GmbH, Germany), and concentration was measured using a Qubit fluorometer (Thermo Fisher Scientific, USA) or Nanodrop ND-1000 UV/Vis spectrophotometer (Nanodrop technologies, USA). The samples were stored at − 20 °C. Sample collection was approved by the Animal Ethics Committee of State Provincial Office of Southern Finland (ESAVI/343/04.10.07/2016).

### Genetic analyses

Genome-wide genotyping was performed in two cases and eight controls using Illumina’s canine HD 173K SNP array at Neogen GeneSeek Operations (Lincoln, NE, USA). The genotype data were filtered using an SNP genotyping call rate of > 95%, an array call rate > 95%, and minor allele frequency of > 0.05. The genotype data are available upon request. After quality control, all 10 samples and 80,835 out of 172,963 SNPs remained for analysis. Quality control and homozygosity mapping were carried out using PLINK 1.07 (Purcell et al. 2007) to identify runs of homozygosity (ROHs). Default parameters in PLINK defining ROH segment and sliding window criteria were used and ROHs overlapping pool of samples were retrieved. The allelically matching ROHs shared by the two cases were selected for further inspection.

Whole-genome sequencing was performed on one Scottish Deerhound at Novogene (Novogen (HK) Company Limited, China) using the Illumina HiSeqX platform with ~ 53 × approximate coverage (paired-end reads, 2 × 100 bp). The genome is available at the NCBI Sequence Read Archive with accession code SRP158843 (BioProject: PRJNA487940). The reads were mapped using the Burrows–Wheeler Aligner (BWA) version 0.7.12-r1039 (Li and Durbin 2009) and the Picard tools (http://broadinstitute.github.io/picard/) were used to sort the mapped reads and to mark duplicates. Canine genome build CanFam 3.1 from Boxer was used as a reference for aligning the sequence reads. Indel realignment, base-quality score recalibration, and variant calling were performed with the Genome Analysis Tool Kit (GATK) HaplotypeCaller 3.5.0 (McKenna et al. 2010). The variants were annotated exploiting Ensembl, RefGene, Broad, and FEELnc (FlExible Extraction of LncRNAs) (Wucher et al. 2017) databases. In addition, we used WGS variant data from publicly available genomes and our other ongoing studies (340 dogs from the DBVDC consortium, see Acknowledgement) as controls in variant filtering (Online Resource 1). To identify potential causative variants, we performed variant filtering with our in-house variant database built and queried using Genotype Query Tools (Layer et al. 2016). CSC computational facilities, a Finnish computational hub, were utilized in the sequencing data analyses (http://www.csc.fi).

The canine *SGK3* mRNA and protein reference sequences were NM_001190428 and NP_001177357.1, respectively.

### Variant screening

The known *SGK3* variant associated with hairlessness in American Hairless Terriers was screened as described in Parker et al. (2017). The candidate variant in *SGK3* was genotyped in a total cohort of 66 SD and 91 IWs using standard PCR and Sanger sequencing to ascertain the association of the variant with the disease. IW breed was selected for the variant screening, because SD was used for the recovery of IW when the breed was re-created, and therefore, IWs are closely related to SDs. The following primer pair was used: 5′-GGTCTCAGTGGGTAGGAGTG-3′ and 5′-GAGCCAGCCAGACACCCT-3′. The primers were designed with the Primer 3 software (Koressaar and Remm 2007; Untergasser et al. 2012), and the amplified PCR products were sequenced with a capillary sequencer at the Institute for Molecular Medicine Finland core facility (FIMM, Technology Centre, University of Helsinki, Helsinki, Finland). The sequences were analyzed using the Sequencher 5.3 software (GeneCodes, USA).

## Results

**Fig. 1:**
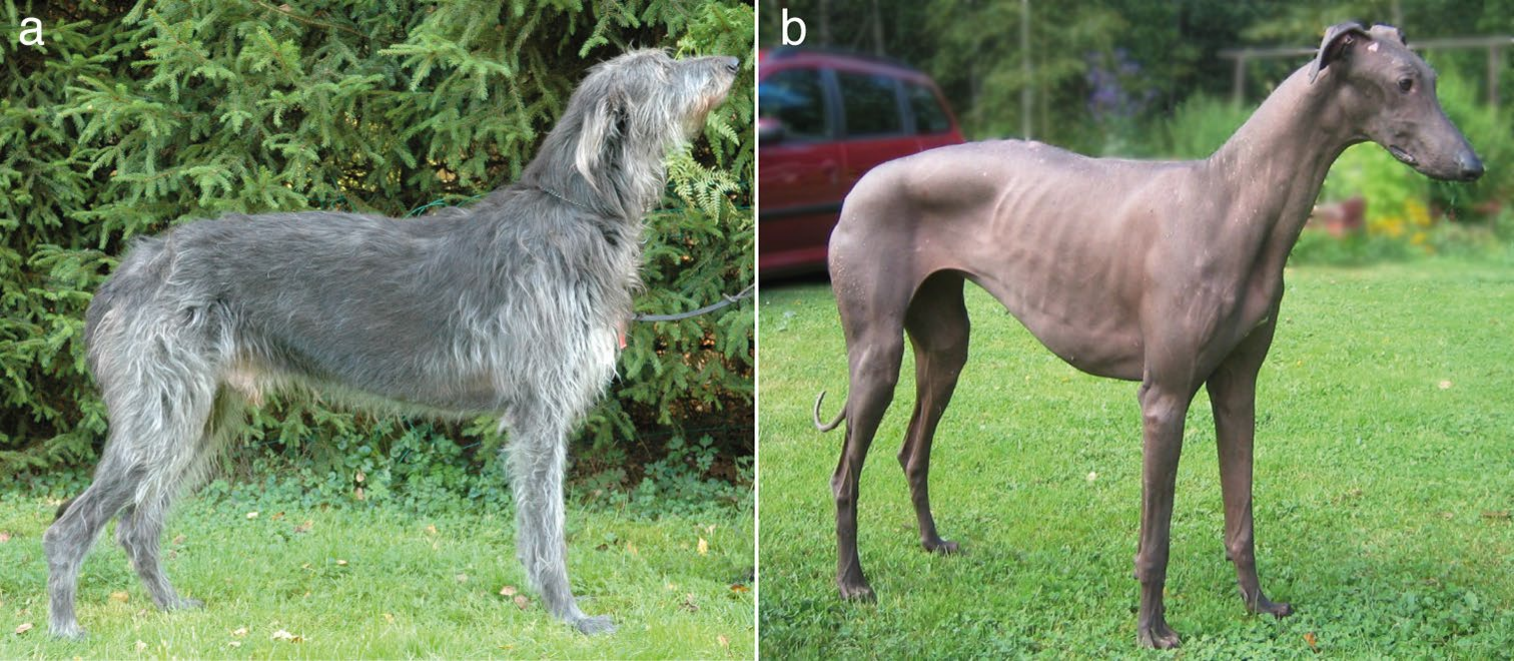
**a** An adult male Scottish Deerhound with normal hair. **b** An adult female Scottish Deerhound demonstrating the hairless phenotype. Photographs used with permission from Antti Salmi (**a**) and Eeva-Kaisa Rantala (**b**)

To explore the genetic background of hairlessness in SDs, we collected DNA samples from 66 dogs, including two hairless dogs (male and female) that were born in a litter of five puppies to parents with normal coats. The affected puppies were born with sparse hair, but lost it completely within the first 2 months of life (Fig. 1). Since the SD’s phenotype resembles the hairless trait in AHTs, the *SGK3* was a plausible candidate gene. We genotyped the known *SGK3* variant, but both of the hairless dogs were homozygous for the wild-type allele, suggesting the existence of a novel genetic cause.

To find the novel cause, we first carried out a genome-wide analysis of two affected and eight unaffected dogs including two obligate carriers using Illumina’s HD 173 K SNP array to map the locus. Homozygosity mapping revealed 12 case-specific regions of continuous allelic homozygosity (Online Resource 2), including a region that spanned the exon 29 of the *SGK3* gene. In parallel, we performed a high coverage (read depth ~ 30 ×) whole-genome sequencing in one hairless dog. Variants (SNVs and indels) were called using CanFam3.1 Boxer genome as reference and filtered against 340 control genomes from various breeds (Online Resource 1) assuming an autosomal recessive disease model and a breed-specific founder mutation in the SD breed. This analysis resulted in the discovery of 45 exonic or splicing variants with a possible effect on protein level (Online Resource 3). However, only two variants were located within the regions of shared allelic homozygosity and were both on chromosome 29: a nonsynonymous change in zinc-finger homeobox 4 (*ZFHX4*) gene and a frameshift insertion in SGK3. *ZFHX4* has an unknown function, but it has been suggested as a candidate gene for ptosis (McMullan et al. 2002) and to be involved in neural and muscle differentiation based on the expression pattern (Hemmi et al. 2006). Due to the unlikely role of *ZFHX4* in hair follicle development, the variant in *SGK3* remains as the most plausible candidate for hairlessness in SDs. SGK3 has been previously associated with hairlessness in mice and dogs.

**Fig. 2:**
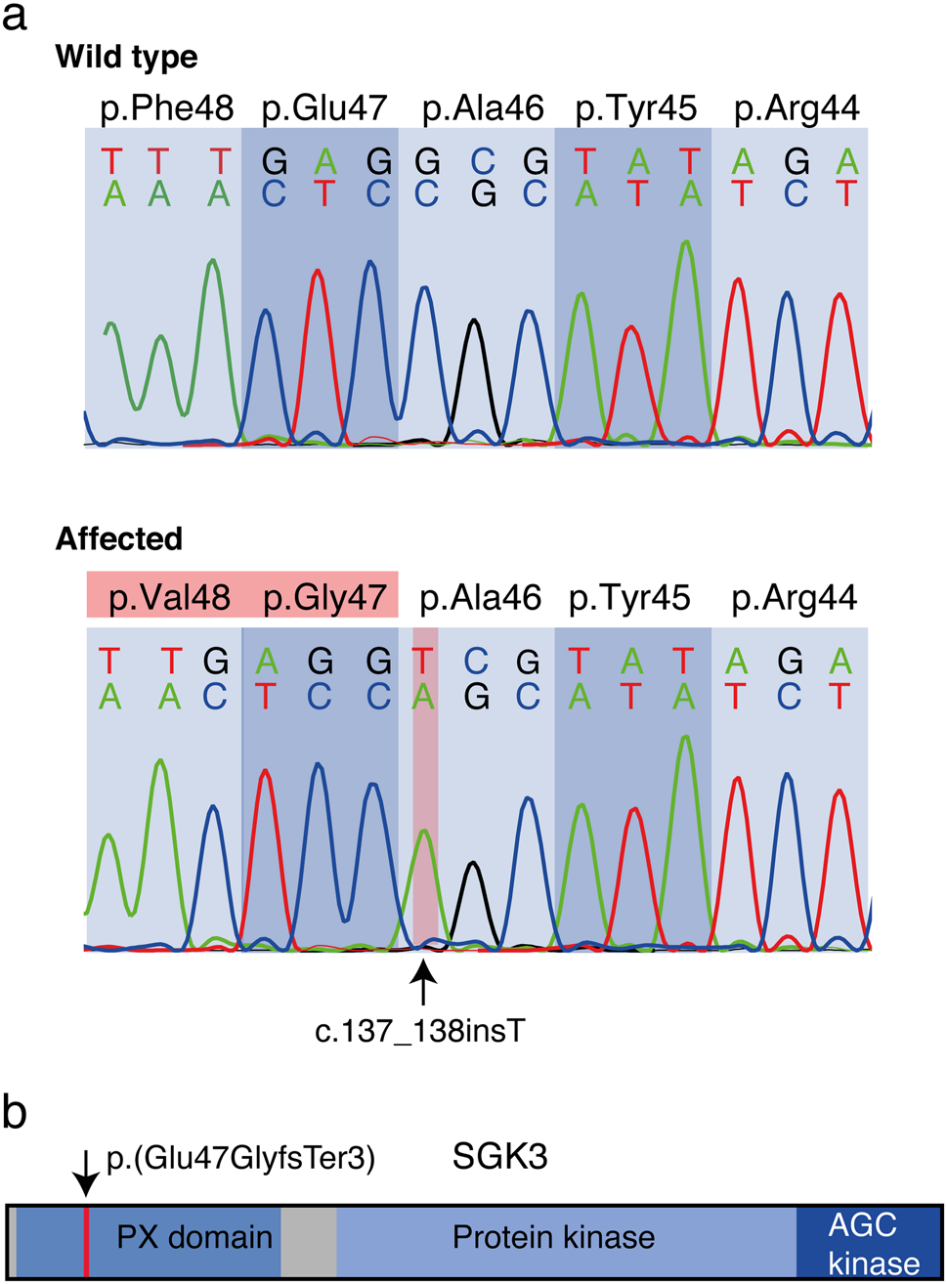
The *SGK3* sequence flanking the variant site and an overview of the protein structure. **a** Sequence chromatograms illustrating the homozygous wild-type allele and the identified *SGK3* variant (c.137_138insT) predicted to result in a frameshift with an early translation termination site. **b** The schematic overview of the SGK3 protein demonstrates the position on the altered amino acid p.(Glu47GlyfsTer3). The predicted truncation removes the majority of the protein including part of the phox homology domain (PX domain), the protein kinase domain, and the conserved AGC-kinase C-terminal domain (AGC-kinase) containing two phosphorylation sites

The identified *SGK3* variant is a 1-bp insertion in exon 2 (c.137_138insT) and predicted to result in a frameshift with an early translation termination site, p.(Glu47GlyfsTer3) (Fig. 2). Due to the very early position of the premature stop codon, the transcript is most likely destroyed by nonsense-mediated mRNA decay. However, even if the protein was translated, it would be severely truncated containing only the first 49 out of 490 amino acids (Fig. 2b).

To evaluate the segregation of the variant with the trait, we genotyped the *SGK3*:c.137_138insT variant (Fig. 2a) in a cohort of Scottish Deerhounds (*n* = 66) containing two affected dogs, two unaffected dogs, which had produced affected progeny, and 62 other unaffected dogs from our biobank. Both affected dogs were homozygous for the variant and the two obligate carriers were heterozygous, while the rest of the dogs were either heterozygous (*n* = 6) or homozygous for the wild-type allele (*n* = 56). These results demonstrate a full segregation of the variant with the disease and indicate a 12% carrier frequency in the studied cohort. We screened the variant also in a related breed, Irish Wolfhound (*n* = 91), but did not find any carriers, suggesting a breed-specific variant in SD population.

## Discussion

We described here a novel recessive risk allele in *SGK3* associating with juvenile alopecia in Scottish Deerhounds. The affected puppies lose their hair during the first few weeks of life which becomes permanent. The phenotype is comparable to the hairlessness in AHTs (Parker et al. 2017), and therefore, it was not surprising to find the defect in the same *SGK3* gene, although with different variants. Both SGK3 variants, p.Glu47GlyfsTer3 in SD and p.Val96GlyfsTer50 in AHT, lead to a very early truncation and likely a complete loss-of-functional proteins.

SGK3 belongs to a serine/threonine protein kinase superfamily whose members are involved in several cellular functions such as cell growth, proliferation, and migration (Tessier and Woodgett 2006). Mice with spontaneous and targeted loss-of-function (LoF) variants in *Sgk3* have defective hair follicle development and abnormal hair cycle, demonstrating the essential role of the protein in these processes (Masujin et al. 2004; McCormick et al. 2004; Alonso et al. 2005). Sgk3 is quite ubiquitously expressed with proposed roles in other tissues such as in renal function by regulation of podocyte integrity and function (Peng et al. 2018).

Canine studies with two spontaneous hairless alleles in *SGK3* support the previous studies in mice and clearly demonstrate how defective SGK3 results in hair loss and alopecia. Human hair disorders can be non-syndromic or in combination with other clinical conditions. Variants in large number of genes have been discovered encoding various types of proteins related to functions in transcription, signal transduction, cell–cell adhesion, lipid metabolism, and cell cycle (Duverger and Morasso 2014). These proteins have essential roles in hair follicle morphogenesis, structure, and regeneration. *SGK3* has not been linked to human hair disorders, but remains as a strong candidate, particularly for congenital hypotrichosis and early hair loss with a monogenic defect. Analysis of the updated version of the gnomAD database (Lek et al. 2016) for *SGK3* variants did not reveal any homozygous LoF variants and there was only one observation of a homozygous missense variant. However, the data did not provide statistical evidence for LoF intolerance (expected/observed LoF ratio 0.5), and given the phenotype in dogs and mice is limited to only hair growth abnormalities, it would be surprising if the gene was required for survival in human. Therefore, it is reasonable to expect to find similar phenotypes also in human.

In conclusion, our study uncovers the second hairless allele in *SGK3* in dogs and highlights its role in hair follicle biology, while defects in *SGK3* are associated with early onset hair loss in murine and canine models. *SGK3* represents an excellent candidate gene for human alopecia and should be screened for deleterious variants in human patients. The SD breed will benefit from genetic testing of the *SGK3* variant to control for this undesired trait in this breed.

## Electronic supplementary material

Below is the link to the electronic supplementary material.

Supplementary material 1 (XLSX 11 kb)

Supplementary material 2 (XLSX 9 kb)

Supplementary material 3 (XLSX 13 kb)
